# Deep learning-based control framework for dynamic contact processes in humanoid grasping

**DOI:** 10.3389/fnbot.2024.1349752

**Published:** 2024-02-28

**Authors:** Shaowen Cheng, Yongbin Jin, Hongtao Wang

**Affiliations:** ^1^Center for X-Mechanics, Zhejiang University, Hangzhou, China; ^2^ZJU-Hangzhou Global Scientific and Technological Innovation Center, Zhejiang University, Hangzhou, China; ^3^State Key Laboratory of Fluid Power and Mechatronic System, Zhejiang University, Hangzhou, China; ^4^Institute of Applied Mechanics, Zhejiang University, Hangzhou, China

**Keywords:** underactuated, anthropomorphic hand, humanoid grasping and manipulation, dynamic process, deep learning, sim to real

## Abstract

Humanoid grasping is a critical ability for anthropomorphic hand, and plays a significant role in the development of humanoid robots. In this article, we present a deep learning-based control framework for humanoid grasping, incorporating the dynamic contact process among the anthropomorphic hand, the object, and the environment. This method efficiently eliminates the constraints imposed by inaccessible grasping points on both the contact surface of the object and the table surface. To mimic human-like grasping movements, an underactuated anthropomorphic hand is utilized, which is designed based on human hand data. The utilization of hand gestures, rather than controlling each motor separately, has significantly decreased the control dimensionality. Additionally, a deep learning framework is used to select gestures and grasp actions. Our methodology, proven both in simulation and on real robot, exceeds the performance of static analysis-based methods, as measured by the standard grasp metric *Q*_1_. It expands the range of objects the system can handle, effectively grasping thin items such as cards on tables, a task beyond the capabilities of previous methodologies.

## 1 Introduction

The advancement of humanoid robots critically hinges on the essential capability of anthropomorphic hands, enabling them to interact with the environment in a way similar to human behavior. While Robotic Grasping and Manipulation Competition (Falco et al., 2018) has demonstrated the progress made in this field, but robustly grasping arbitrary objects with a anthropomorphic hand remains an open problem (Hodson, 2018). Although research has been done on robot grippers with few degrees of freedom (DoF) (Fang et al., 2023), their inherent constraints in size and degrees of freedom hinder their ability to perform versatile grasping and manipulation. In contrast, the anthropomorphic hand is considered the most ideal universal end effector in a human-centered environment due to its potential to grasp objects with arbitrary shapes and uneven surfaces (Billard and Kragic, 2019). Therefore, developing effective control frameworks for the anthropomorphic hand is crucial for a variety of applications, ranging from manufacturing to service to security.

In recent years, advancements in anthropomorphic hand have been remarkable, and the demonstrated potential of fully-actuated robot hands has significantly influenced the field of manipulation (Andrychowicz et al., 2020). However, the high dimensionality of the search space for fully-actuated hands often results in low learning efficiency and unnatural motions (Mandikal and Grauman, 2021). Another option is underactuated robot hand (Catalano et al., 2014). Thanks to its light weight, compact structure and shape adaptability, it is also widely used in grasping task. But the configuration of the fingers is uncertain when they come into contact with an object, posing a significant challenge for control (Yao et al., 2009).

Grasp synthesis is usually viewed as a constrained nonlinear optimization problem (Miao et al., 2015), which can fall into a local optimal solution due to the high-dimensional space. The position on the contact surface between the object and the table surface is often out of reach, which cannot be ignored as a constraint. Consequently, statics analysis methods prove inadequate for grasping slender objects like cards and coins. Various distinctive gripper structures and control strategies have been proposed, including the utilization of wide fingertips for scooping (Babin and Gosselin, 2018) and prying grasp (Zhang et al., 2022). Additionally, leveraging environmental fixtures (Tong et al., 2020) and the edges of a table (Eppner et al., 2015) has been suggested to achieve successful grasping. Hence, there is a drive to formulate a anthropomorphic hand grasping strategy capable of adeptly handling thin objects.

**Figure 1:**
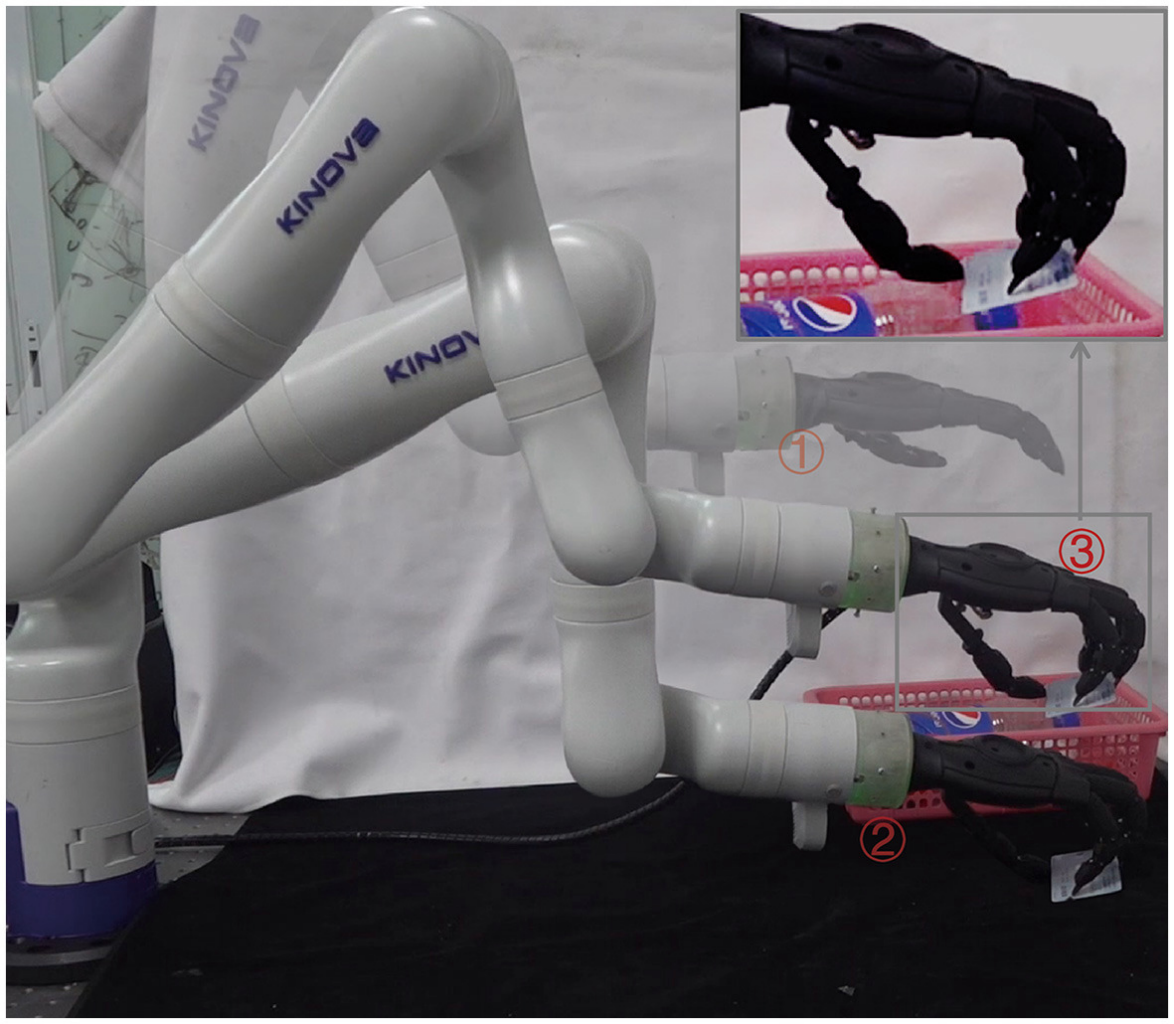
Successful grasp of a thin card from a table surface using our proposed method. The grasp is achieved using a Gen3 Lite robot with a custom underactuated anthropomorphic hand, and is guided by an RGB image captured by the camera mounted on the top of the table.

Inspired by the success of human grasping (Tong et al., 2020), a grasping control strategy is proposed that utilizes the dynamic process between the hands, objects and the environment. This approach allows for any contact point to be accessed on any surface of the object, thereby overcoming the inherent limitation of static analysis. The effectiveness of the proposed grasping control strategy is validated through experiments conducted in a dynamic simulation engine MuJoCo (Todorov et al., 2012) and on the real robot. The dynamic process enables thecontroller to grasp a wide range of objects, including thin cards from table surface, as shown in Figure 1. According to the grasp quality metric *Q*_1_, our method has higher grasp quality compared to methods based on statics analysis.

## 2 Related work

Grasp synthesis is a critical component of autonomous grasping strategies, aiming to attain stability when grasping any type of objects. This topic is approached from two distinct points of view: enhancements in hardware technology and the progress of algorithmic methodologies.

### 2.1 Mechanical design

Mechanical design has been a key strategy for researchers who are striving to imitate the human ability to grasp (Piazza et al., 2019). However, the complex structure of the human hand, with its high DoF and integration of perception systems, poses a significant challenge for robot replication (Hodson, 2018). Additionally, both the delicate structure (Chalon et al., 2010) of human hands is difficult to replicate and the current robot sensors (Xia et al., 2022) lack the precision to imitate human grasping. Researchers have also explored achieving grasping capabilities with non-anthropomorphic hands, such as the combination of suction and two-finger/three-finger gripper. Improvements have been made to the structure and transmission mode of these grippers, such as adopting the underactuated tendon-driven method (Stuart et al., 2017) and incorporating continuous rotation capability of rolling fingertips in some work (Yuan et al., 2020). Grasping in a manner similar to human can adapt to items of arbitrary shapes in daily life. To address the design difficulties of replicating human hands, underactuated hand with tendon driven (Shirafuji et al., 2014) has become increasingly popular as end effectors for humanoid robots (Diftler et al., 2012).

### 2.2 Analytic methods

Analytical methods usually formulate grasp synthesis as a nonlinear constrained optimization problem. During the grasping process, the object and hand's velocity and acceleration are small, enabling the simplification of the analysis through a quasi-static method (Bicchi and Kumar, 2000). *Graspit!* (Miller and Allen, 2004), being the preeminent tool within the community for executing grasps, leverages quasi-static analysis to maximize grasp quality. However, this method can be time-consuming for grasp planning. To achieve real-time behavior synthesis, some researchers have attempted to use MPC methods to realize object grasping and manipulation (Kumar et al., 2014). However, real robot tests have revealed sensitivity to modeling errors. In general, analytical methods are only suitable for accurately modeling geometries and manipulators. Some objects such as thin cards are limited in their grasping potential as contact points cannot be planned on the contact surface of the card and table due to environmental constraints that can only be lifted through dynamic processes.

### 2.3 Data-driven methods

With the advancement of simulators and deep learning, a data-driven approach to the grasp synthesis holds great promise (Bohg et al., 2014). Researchers have presented a deep learning architecture for detecting grasps (Lenz et al., 2015), and techniques such as adding noise (Mahler et al., 2017) and domain randomization (Andrychowicz et al., 2020) have been proposed to achieve the transfer from simulation to the real robot. In the framework of deep learning, various methods have been widely used. Supervised learning has been used to select the best candidate grasps (Mahler et al., 2019), while learning from demonstration has been used to achieve specific tasks (Rajeswaran et al., 2018). For parallel gripper represented by Dex-Net 2.0 (Mahler et al., 2017), the success rate can reach 99%. However, for anthropomorphic hand, equipped with a high DoF, presents a significant challenge. Even without addressing the complexities of object dynamics, exploring a reasonable grasp action for a high DoF dexterous hand remains a challenging task (Roa et al., 2012). The difficulty amplifies further when dealing with objects of uncertain shapes (Li et al., 2016). Additionally, reinforcement learning techniques have been leveraged to achieve remarkable performance in tasks deemed challenging for humans (Chen et al., 2022). While significant progress has been made in studying specific manipulation tasks with anthropomorphic hands, their high-dimensional search space limits their performance in grasping objects of any shape, particularly thin objects (Liu et al., 2019). In recent years, some work have utilized RL based on synergies (Liang et al., 2022) to accomplish high DoF dexterous hand grasping and manipulation, showcasing promising prospects. However, it requires long training times and the success rate is still lower than parallel gripper. Therefore, we adopt a supervised learning method. Utilizing objects with simple shapes as the training dataset allows us to derive a controller in approximately 10 min. In addition, we explore more grasp actions using dynamic data, thereby improving the success rate. This article based on grasp dynamic data and synergies methods, seeks to achieve the grasping of objects of any shape with a high DoF hand.

## 3 Methodology

This section delineates the method employed to achieve stable grasp with a custom anthropomorphic hand (Bin Jin et al., 2022). The hand is a 6 active DoF underactuated hand driven by twisted string. The flexion of the fingers is driven by a tendon, while the thumb's abduction-adduction is directly driven by a motor. This type of underactuated hand has excellent shape adaptability, allowing us to implement open loop control of the robot hand. The core of our approach involves the entire dynamic process, the reduction of space dimensionality through gestures, and the metric for evaluating the grasp action.

### 3.1 Definitions

**Figure 2:**
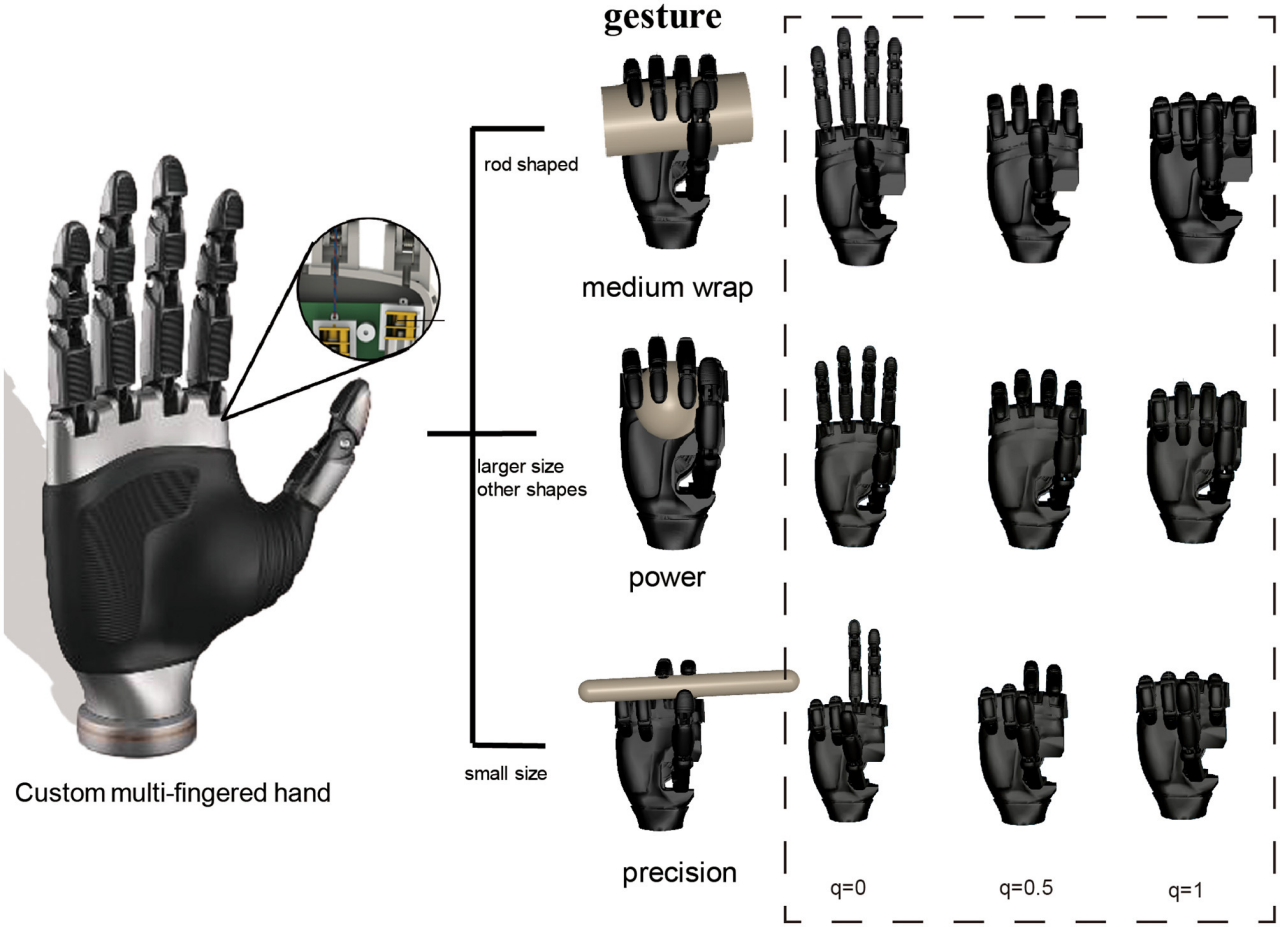
The gesture of the custom anthropomorphic hand is depicted. The underactuated hand is of similar size to a human hand and consists of 6 active degrees of freedom and 15 passive degrees of freedom. We artificially defined three gestures: medium wrap, power, and precision. Objects exhibiting cross-sections less than 4 cm, characterized by the longest side length, are identified as precision grasp. Conversely, they are classified as power grasps or medium wrap grasps, contingent upon their shape. The method of specifying gestures can be seen as offering multiple types of end effectors for grasping objects with different shapes. The correlation between the degree of control for every gesture and the configuration of the finger is elucidated on the right side of the figure. A detailed numerical relationship is provided in Table 1.

Gesture *T*: A single variable selected from three specific gestures denoted by *T*_1_, *T*_2_, and *T*_3_ as shown in Figure 2. Santello et al. (1998) indicates there is a high correlation between the angles of all finger joints. This finding suggests that by using low-dimensional gestures, the complexity of finger joint space can be significantly reduced. And the paper by Cutkosky (1989) depicts that human hands predominantly employ power and precision grasp for object. For the circular object, the generalized freedom of gestures can cover both power and precision gestures. For the prismatic object, we have separately chosen a gesture for power and precision. As a result, the three gestures we have defined—medium warp, power, and precision—are capable of grasping objects of various shapes and sizes.

**Table 1:** Numerical relationship between gesture control amount and finger joint angle.

	**Index**	**Middle**	**Ring**	**Little**	**Thumb (aa)**	**Thumb (fe)**
Medium wrap	*q*×1.57	*q*×1.57	*q*×1.57	*q*×1.57	1.8	*q*×0.3
Power	*q*×1.57	*q*×1.4	*q*×1.4	*q*×1.57	1.4	*q*×0.4
Precision	*q*×1.57	*q*×1.57	0	0	1.4	*q*×0.2

**Figure 3:**
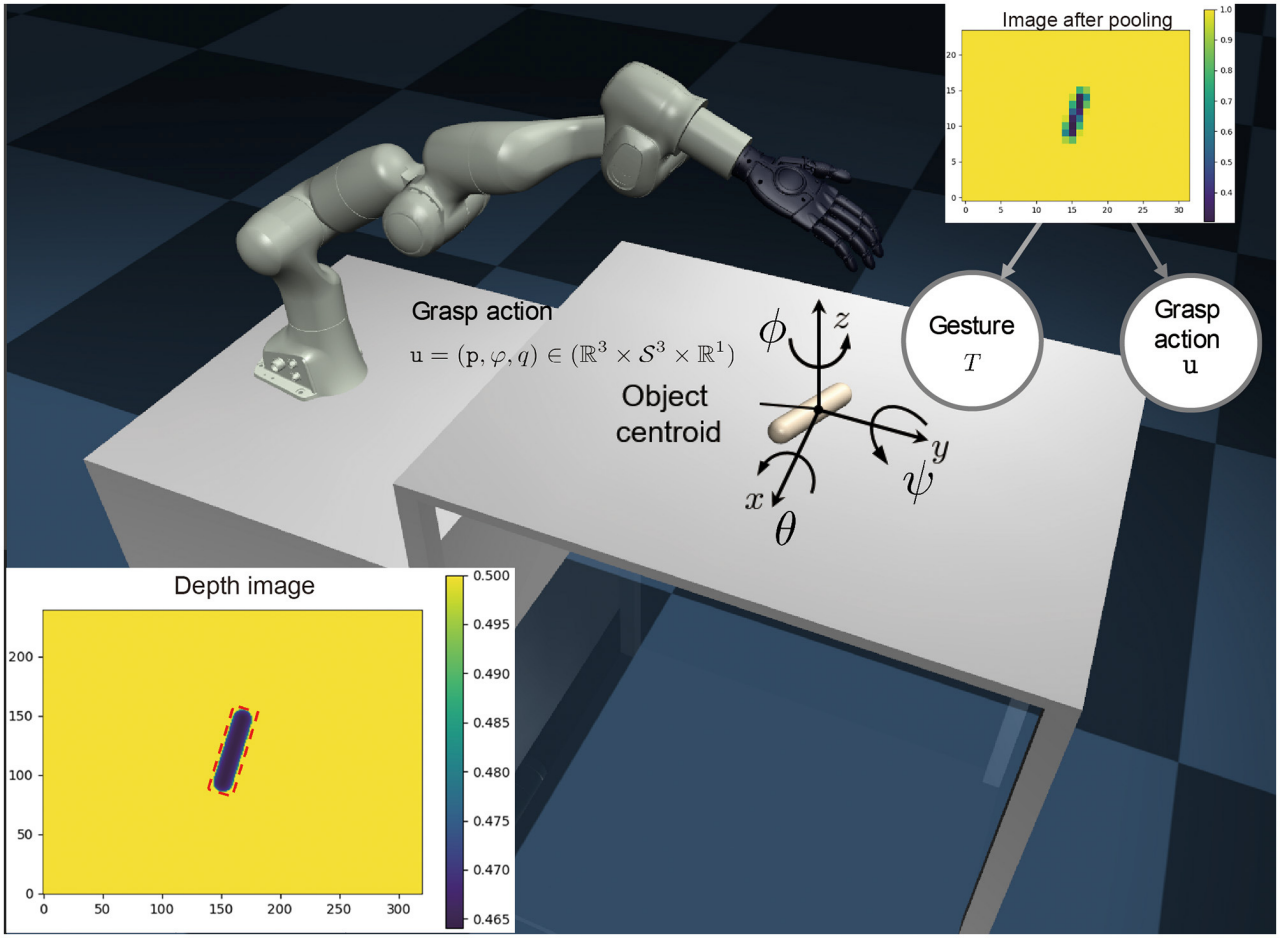
Overview of our approach. A monocular depth image is generated from 3D meshes captured by a camera positioned above the table (As shown in in the **lower left corner**). This image is crucial in estimating the centroid and the rotation ϕ_0_ within the plane. Our objective is to select the gesture *T* and grasp action *u* based on the depth image *y* (exemplified in the **upper left corner**) after maxpooling. The three types of gestures *T* are illustrated in Fig.2. The grasp action *u* is defined by the relative position of the hand with respect to the object centroid *p* ∈ *R*^3^, the orientation in Cartesian space, and the close ratio of the hand *q* ∈ *R*^1^.

Grasp action *u*: A tuple $u=\left(p,\phi ,q\right)\in \left({\mathbb{R}}^{3}\times S^{3}\times {\mathbb{R}}^{1}\right)$, where *p* denotes the position of the hand relative to the centroid of the object, ϕ denotes the orientation of the hand, and *q* denotes the generalized degrees of freedom of the selected gesture. These variables are illustrated in Figure 3.

Depth image *y*: A representation of the object in the form of a depth image $y=R_{+}^{H\times W}$ with height *H* and width *W*. Grasp quality metric *Q*: A metric used to evaluate the stability of the grasping, defined by the equation:


(1) $$\begin{array}{c} Q=2\left(e^{-x}-0.5\right)\in \left[-1,1\right] \end{array}$$

where *x* represents the variance of the object displacement while applying a random external force after the grasp is completed, as proposed in Ferrari and Canny (1999).

### 3.2 Problem statement

The challenge of planning a robust grasp for a single rigid object can be addressed by selecting the most suitable gesture *T* and grasp action *u*. This article aims to find the gesture *T* and grasp action *u* that maximize the grasp quality *Q*, which can be inferred from the depth image *y*.

The gesture *T* can be selected from the set *T* = {*T*_1_, *T*_2_, *T*_3_} using a gesture selection neural network. The optimal gesture *T* is determined by the equation:


(2) $$\begin{array}{c} T^{*}=\underset{T^{*}\in T}{\arg\max}f\left(y,T\right) \end{array}$$

The grasp action *u* can be selected from a set of candidates by a grasp quality evaluator, which maps the grasp action *u* to a quality metric *Q*. The optimal grasp action *u* is determined by the equation:


(3) $$\begin{array}{c} u^{*}=\underset{u^{*}\in U}{\arg\max}Q\left(u,y\right) \end{array}$$

The main objective of this paper is to develop a robust grasp planning system that successfully grasps an object based on its depth image *y*.

### 3.3 Method

The grasping problem is approached by dividing it into two sub-problems. Firstly, a gesture selection neural network (GSNN) is trained as a classification problem to determine the appropriate gesture T^*^. Secondly, a dynamic grasp quality neural network (DGQNN) is trained to map the grasp action *u* and the quality metric *Q*. Both evaluators, the GSNN and DGQNN, are trained using supervised learning. Consequently, the selection of gestures and grasp actions becomes decoupled, utilizing independent datasets to optimize their respective performances.

#### 3.3.1 Gesture selection neural network

To reduce the computational complexity of analyzing the high-dimensional degrees of freedom of a custom anthropomorphic hand, three gestures are manually defined based on grasp taxonomy research: power, intermediate, and precision. Figure 2 illustrates that these gestures can effectively cover objects with basic shapes such as box, sphere, cylinder, etc. Most objects in daily life can be approximated to these fundamental shapes. Our method refers to the neural network framework of LeNet-5 (LeCun et al., 1998) and utilizes the convolutional neural network structure depicted in the upper part of **Figure 5** to train the GSNN. Approximately 60,000 data are generated to train the gesture evaluator. For example, we manually designed the medium wrap gesture for rod-shaped objects, and power and precision gestures are distinguished by the size of the object's longest side length. Those with cross-sections greater than 4 cm had the longest side length are labeled as a power grasp, while those with the longest side length less than 4 cm are labeled as a precision grasp. The dataset is split into training and test sets, the GSNN attained 99% accuracy on the test dataset.

#### 3.3.2 Grasp action dataset

Collecting dynamic data is essential for learning a grasp quality evaluator to evaluate the performance of grasping. The intuitive approach to evaluating grasp quality is based on the maximum wrench magnitude over all possible directions (Ferrari and Canny, 1999). An advanced dynamics simulation engine MuJoCo (Todorov et al., 2012) us used to simulate the entire dynamic process of grasp and generate grasp action dataset.

**Figure 4:**
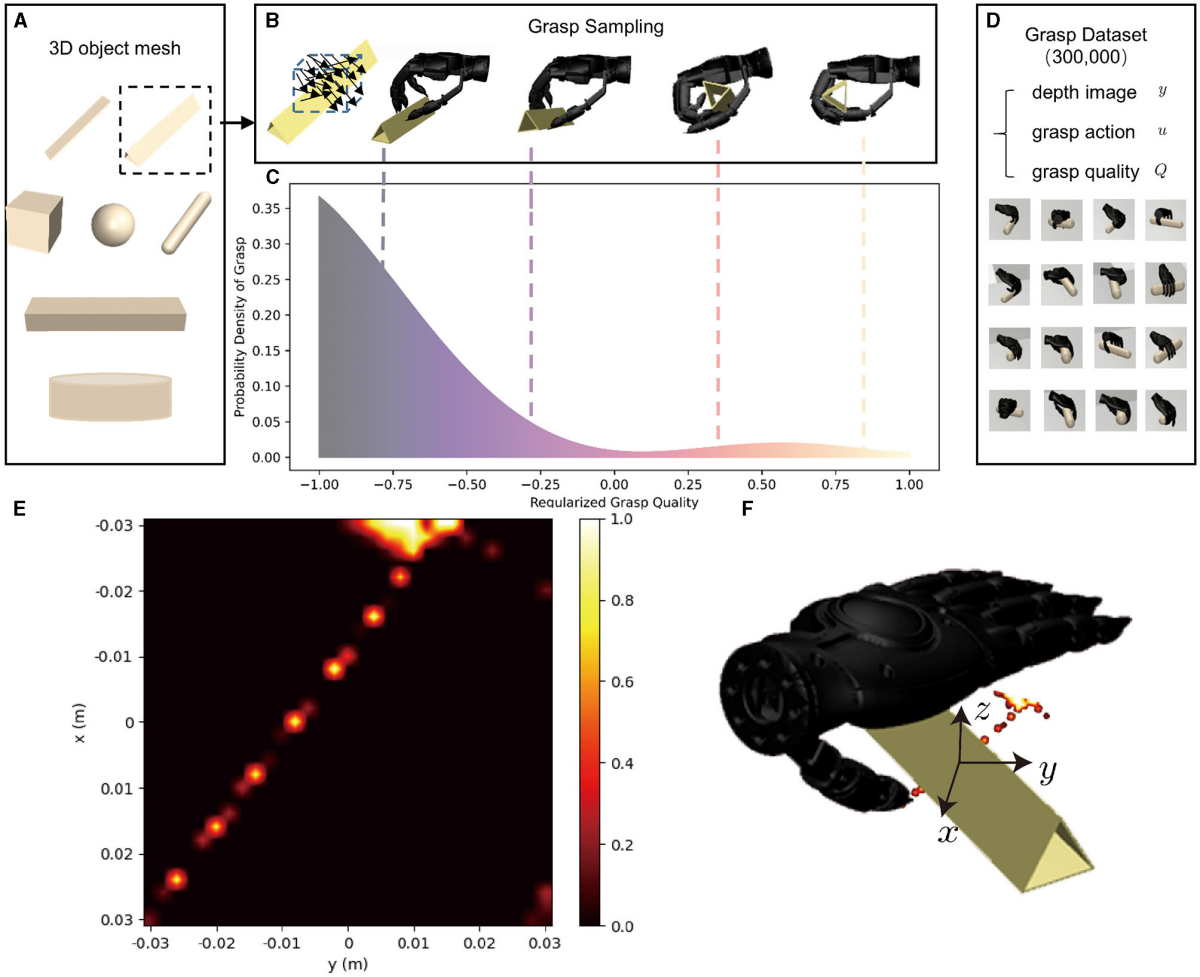
The pipeline for training data generation. In the **(A)**, it is shown that objects in daily life are composed of objects with simple shapes, and the training datasets include seven types of 3D object meshes. In the **(B)**, an object is randomly placed on the table, and its depth image is recorded by the camera above the table. The object's centroid and rotation ϕ_0_ is calculated based on the image, and grasp action is sampled near the centroid. The grasp quality is then evaluated, and its probability density distribution is shown in the **(C)**. The grasp quality distribution for the triangular prism reveals that good grasp occupy only a small part of the entire sampling space. In the **(D)**, it is shown that each dynamic grasp data contains a 32 × 24 depth image *y*, grasp action *u*, and the corresponding quality *Q*. There are nearly 300,000 entries in the full dataset. **(E)** This is a heat map indicating the grasping success rate of triangular prism with a rotation of ϕ_0_ plotted on the xy plane at *z* = −0.8cm, ψ = 0rad, θ = 0.3rad, ϕ = 0.5rad, within a range of [–3 cm, 3 cm]. **(F)** Visualization of successfully grasped positions in the simulation environment.

At the initiation of each grasping attempt, an object is randomly selected from the seven basic shape types as shown in Figure 4A, and placed on the table with random size and location. Subsequently, a depth image *y* of the object is captured by a camera directly positioned above the table. The object's centroid and rotation ϕ_0_ is calculated based on the image, and the image undergoes downsampling to a resolution of 32 × 24 is saved through max pooling, which is deployed to retain the object's edge information to the greatest extent feasible. Next, the grasp action *u* is uniformly sampled near the object's centroid in the space of *x, y, z* ∈ [−3cm, 3cm], ψ ∈ [−0.2rad, 0.2rad], θ ∈ [−0.1rad, 0.6rad], ϕ ∈ [ϕ_0_ − 1.57rad, ϕ_0_ + 1.57rad]. After the grasp is completed, the grasp quality *Q* is evaluated based on the ability to resist external forces *f* as indicated by the variance of the slip distance *x* of the object. The pipeline for training data generation is shown in Figure 4. The grasp dataset contains a depth image *y*, a grasp action *u*, and the corresponding grasp quality *Q*. The full dataset contains almost 300,000 samples. As shown in Figure 4B, a triangular prism object as a representation, situated near the centroid, with uniform sampling in the space of *x, y, z* ∈ [−3cm, 3cm], ψ ∈ [−0.2rad, 0.2rad], θ ∈ [−0.1rad, 0.6rad], ϕ ∈ [ϕ_0_ − 1.57rad, ϕ_0_ + 1.57rad]. Notably, only a small part of the grasp actions proved successful, as shown in Figure 4C. We tested the success rate every 2 mm within the range of *x* ∈ [−3cm, 3cm], *y* ∈ [−3cm, 3cm] on the plane, with the parameters *z* = −0.8cm, ψ = 0rad, θ = 0.3rad, ϕ = 0.5rad, ϕ_0_ = 0.5rad being fixed. Each case undergoes 20 trials of grasp to establish the success rate. The heat-map obtained by statistics is shown as Figure 4E, and the corresponding grasp position has been mapped to Figure 4F. Interestingly, the position with the highest success rate of grasp is near *x, y* = (−2.8cm, 1cm), not near the centroid of the object, which is contrary to people's understanding. This is because at these positions, the interaction of the robot hand with the object and the table breaks the limit of the contact surface, thereby achieving successful grasp. Indeed, it is through the utilization of this dynamic data that we are able to break the limitations of the contact surface of object and table surface, resulting in a significantly improved success rate.

#### 3.3.3 Dynamic grasp quality neural network

**Figure 5:**
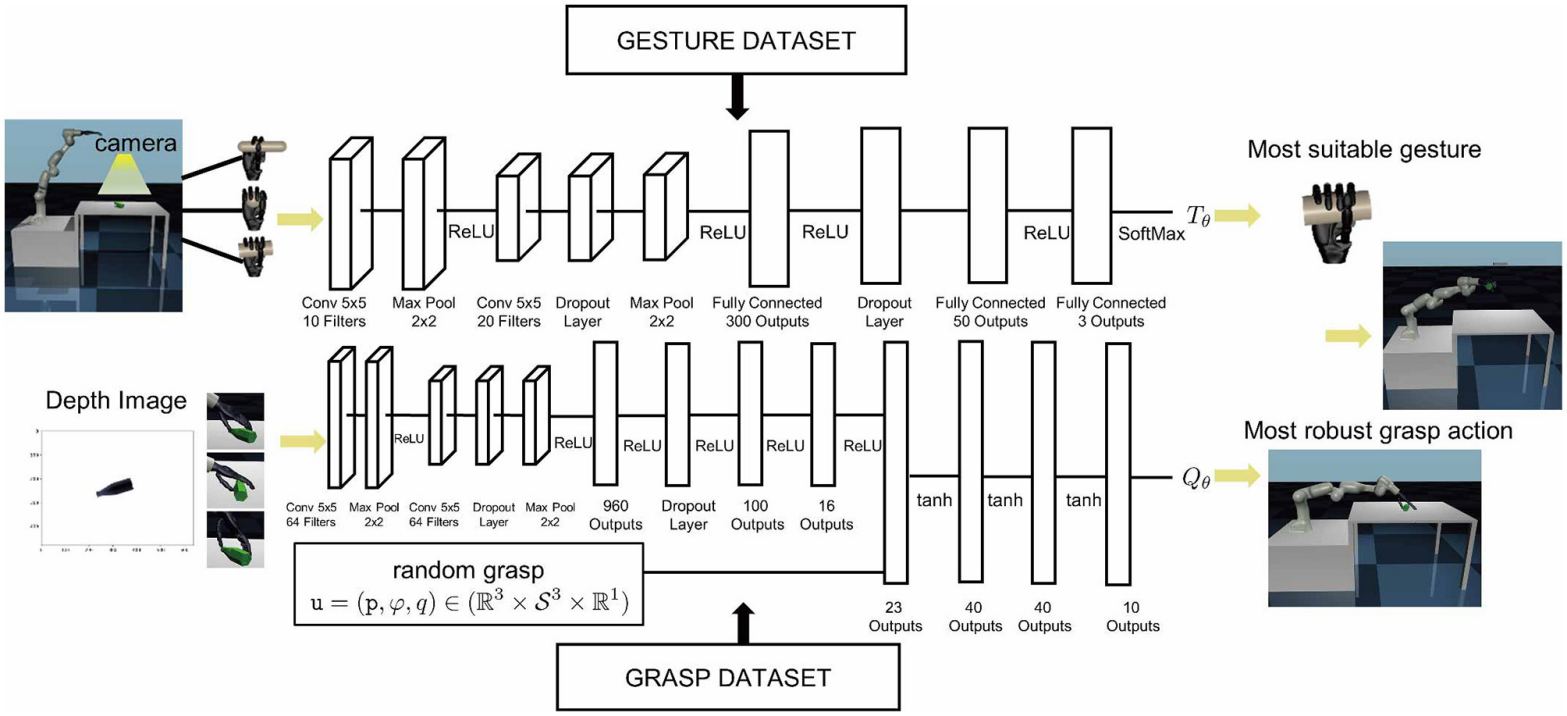
Architecture of the dynamic humanoid grasp. **(Top)** The Gesture Selected Neural Network (GSNN) is trained offline to select the optimal gesture from the depth image. The architecture consists of two convolutional layers in pairs of two separated by ReLU nonlinearities followed by four fully connected layers. **(Bottom)** The Dynamic Grasp Quality Neural Network (DGQNN) is trained offline to predict the quality of candidate grasps from depth images using grasp action dataset. The architecture consists of three convolutional layers in pairs of two separated by ReLU nonlinearities followed by eight fully connected layers and a separate input layer for the grasp. **(Left)** When an object is presented to the robot, a depth camera captures a depth image, and one thousand grasp candidates are generated in the sampling space. **(Right)** The DGQNN rapidly determines the most robust grasp candidate, which is executed with the Gen3 robot.

Once the appropriate gesture is selected, the objective is to determine the optimal grasp action *u*. However, the dynamic grasp dataset in Figure 4C indicates that the number of successful grasps is extremely small, necessitating the establishment of a grasp quality evaluator to fit this data and identify the optimal grasp action *u*. To accomplish this objective, we construct a Dynamic Grasp Quality Neural Network (DGQNN) inspired by the GQ-CNN network (Mahler et al., 2017), as demonstrated in Figure 5 bottom.


(4) $$\begin{array}{c} \theta^{*}=\underset{\theta \in \Theta }{\arg\max}L\left(Q,Q_{\theta }\left(u,y\right)\right) \end{array}$$

**Figure 6:**
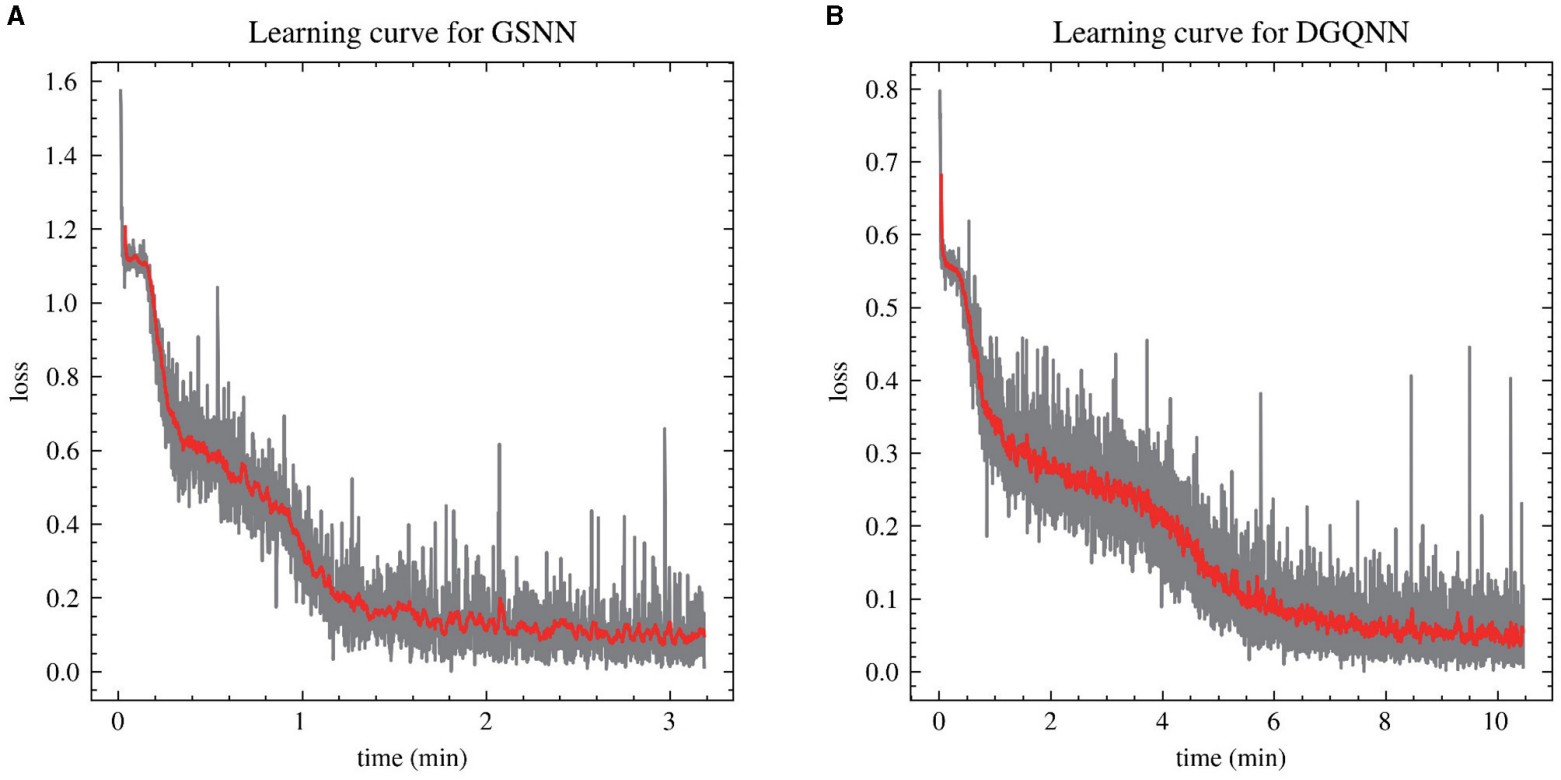
Learning curve for GSNN **(A)** and DGQNN **(B)**.

The DGQNN is defined by the set of parameters θ that represent the grasp quality evaluator *Q*_θ_. The input data undergoes a normalization process before being passed through a series of convolutional layers for image input *y*. Concurrently, the grasp action input *u* is directed through fully connected layers to achieve an estimation of grasp quality denoted by *Q*. The neural network has approximately 60,000 parameters, which are optimized using backpropagation with stochastic gradient descent and momentum. The training configurations of the two networks are shown in Table 2. The neural network is trained using Torch on NVIDIA GTX 1080Ti, and the training can be completed in about 10 min as shown in Figure 6.

**Table 2:** The training configurations of the two networks.

	**GSNN**	**DGQNN**
Learning rate	0.01	0.0001
Batch size	64	100
Number of epochs	30	100
Momentum	0.5	0.9
Dropout	0.5	0.5
Optimizer	SGD	SGD
Loss function	Cross-entropy loss	Cross-entropy loss

## 4 Results

A comprehensive evaluation of grasp performance is conducted in both simulation and real robot, utilizing a custom anthropomorphic hand and the KINOVA gen3 robot. To establish a benchmark for grasp performance, a comparison is made with other approaches for high DoF anthropomorphic hand grasping (Liu et al., 2019, 2020). The results demonstrated that our framework outperformed other methods, as measured by the standard metric (Ferrari and Canny, 1999).

### 4.1 Simulation results

During the grasp planning phase, our primary step entails computing the centroid and rotation ϕ_0_ of the target object utilizing the original depth image. For precision and medium wrap gestures, the hand is need to aligned with the object ϕ_0_. And then the optimal gesture is selected by maximizing the gesture evaluator *f*(*y*) among the gestures. Subsequently, 1,000 grasp actions are sampled uniformly in the space of *x, y, z* ∈ [−3cm, 3cm], ϕ ∈ [ϕ_0_ − 1.57rad, ϕ_0_ + 1.57rad], θ ∈ [−0.1rad, 0.6rad], ψ ∈ [−0.2rad, 0.2rad], near the object's centroid and rotation, and the highest quality grasp action candidate is determined using the grasp quality evaluator $Q_{\theta^{*}}$. A uniform sampling of 1,000 points is conducted in a 6-dimensional space, yielding an average of $\sqrt[{6}]{1000}\approx 3$ points for each dimension. These three points represent the minimum, median, and maximum values of this dimension, covering the entire space. The grasp action policy π_θ_(*y*) = $\underset{u\in C}{\arg\max}$*Q*_θ_(*u, y*) is employed to execute the grasp action *u*, where *C* specifies constraints on the set of feasible grasps such as collisions or kinematic feasibility. By optimizing the gesture *T* and grasp action *u*, this approach enables effective and reliable grasp.

**Figure 7:**
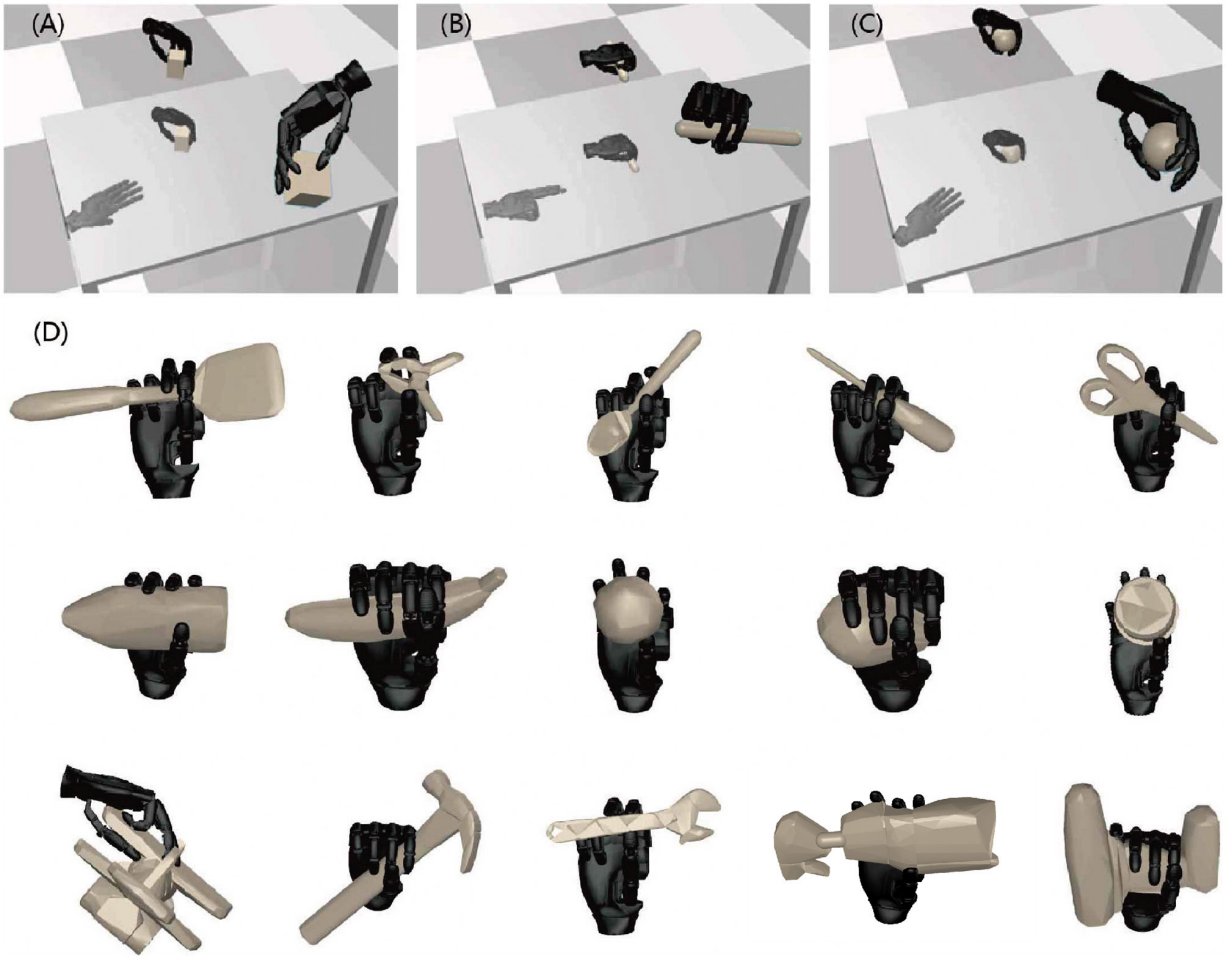
Shows the results of testing the generalization for all objects, none of which were included in the training dataset. **(A–C)** depict the process of object grasping, highlighting its natural and human-like characteristics. It is noteworthy that regardless of the specific gesture design, our method consistently utilizes as many contact points as possible, leading to a significant increase in the grasp success rate. **(D)** shows the grasping results of the objects in the YCB database, demonstrating the generalization capability of our method for objects with complex shapes. The results in the figure are not near the centroid of the object, for example, the airplane. In the figure, some of the grasp positions are not near the centroid of the object, as exemplified by the airplane. This is due to the interaction between the robot hand and the object.

The generalization capability of the method is assessed through grasping tests on objects not encountered during training. Impressively, this method achieves the success rate of 93.4%, successfully grasping thin-shell objects like cards and triangular prisms, which fail with static methods. In Figures 7A–C, regardless of the specific gesture, the control strategy adopts an approach that utilizes as many contact points as possible to grasp objects, significantly increasing the success rate. For instance, in Figure 7B, our initial gesture design aimed to pinch the object using the thumb, index finger, and middle finger. However, during testing, we observed that when dealing with long objects, the ring finger and little finger are employed to provide additional support and hold the object firmly. This adaptability is a direct result of our dynamic process that explores and selects grasping methods with the highest quality, ensuring robust and versatile grasping performance.

In comparison to other High-DoF Gripper planners designed for YCB objects as shown in Table 3 (Liu et al., 2019, 2020), our method demonstrates a superior success rate of 92% in a test involving 50 objects. The definition of a successful grasp is as follows: Initially, the relative position of the object to the wrist is recorded upon the completion of the grasp action by the robot hand. Next, the object is lifted 30cm upwards while maintaining an unchanged wrist pose. Throughout this operation, an external force of *f* ∈ [0, 5*N*] is exerted, its direction randomly sampled within the entire space. Changes in the relative position are recorded. The grasp is label as successful if the spatial variance remains under 1cm. Comparatively, the objects employed for testing are selected from the YCB objects, mirroring those utilized in our baseline comparisons. This is attributed to our method's utilization of a dynamic database and exploration of various grasp actions for establishing contact points in the object's surface, thereby yielding superior grasp quality as shown in Figure 7D. Additionally, our method employs gestures to enhance computational efficiency. The entire process, encompassing image processing, gesture selection, and grasp action selection, requires a total of 0.41s, with 0.02s allocated to gesture selection and 0.39s to grasp action selection, signifying a significant improvement over previous works. While our method successfully addresses the grasping of thin objects, we encountered failures with four objects characterized by oversized shapes and specific grasp position requirements, namely, the Master chef can, pitcher base, pitcher lid, and plate.

**Table 3:** For the 50 YCB objects in the testing set, we compare the predicted quality of grasp poses in terms of the Q1 metric, planning time, success rate for the grasp and success rate for the thin object.

**Method**	***Q*_1_**	**Planning time(s)**	**YCB success rate**	**Thin object success rate**
Ours	0.31	0.41	92.0 %	100%
Liu et al. (2019)	0.23	3	66.0%	0%
Liu et al. (2020)	0.11	/	54.0%	0%

### 4.2 Real robot experiments

**Figure 8:**
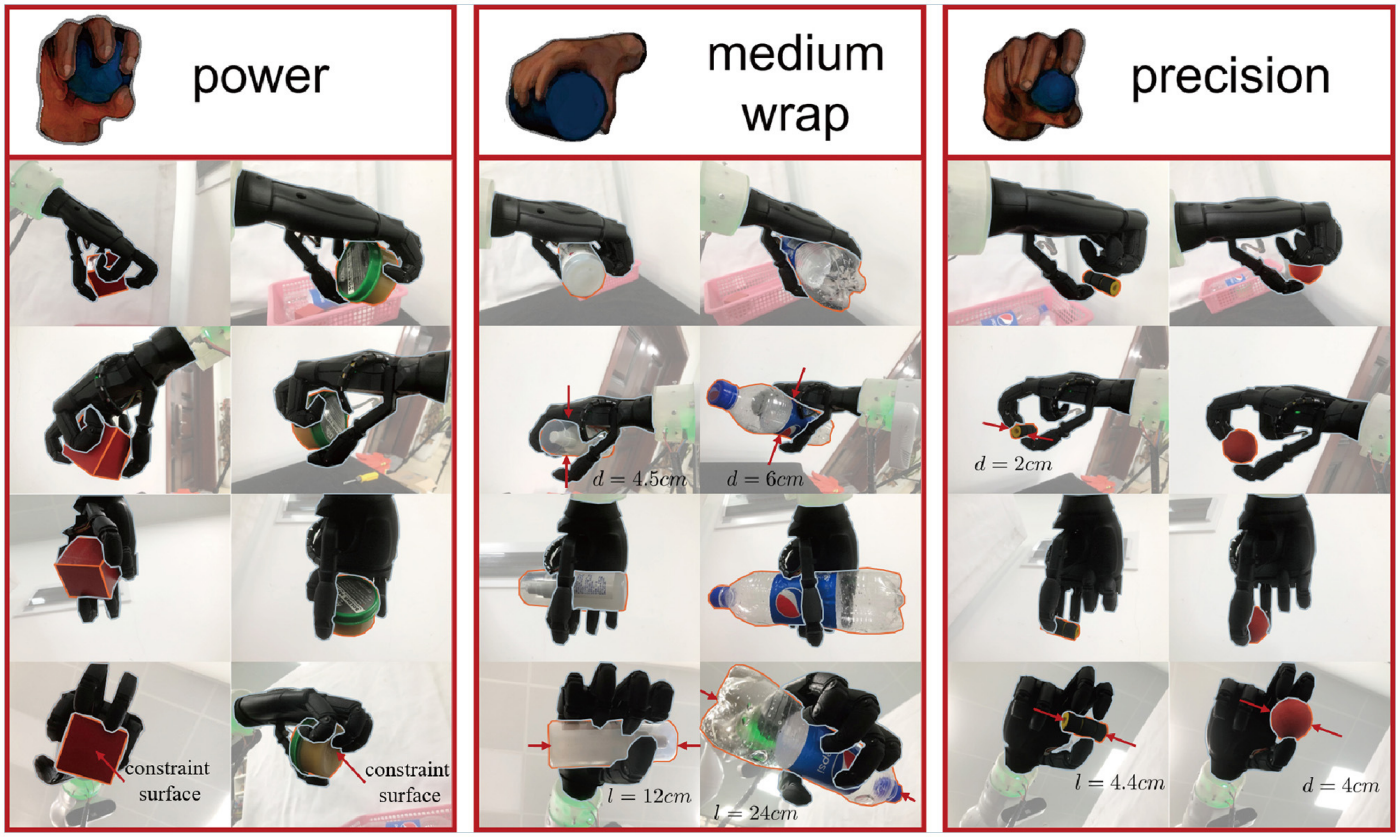
Shows the test set of six objects of different shapes and sizes used to evaluate the generalization performance of the controller. The experiments on real robot demonstrate that all the previously defined gestures are effectively utilized for grasping and achieve good grasp quality.

Experiments are also conducted on a real robot comprising an underactuated custom anthropomorphic hand, a KINOVA gen3 robot, and an RGBD camera. The gesture *T* and grasp actions *u* are executed using the previously mentioned strategy, with the input depth image of the object. In the actual grasping experiment, the hand executed a predetermined gesture, and after the KINOVA gen3 robot arrived at the specified pose, the hand is closed based on the output generalized degrees of freedom to complete the grasping. As demonstrated in Figure 8, our approach successfully transferred to reality. The gestures are effectively applied to objects of various sizes and shapes, with precision gesture employed to grasp small objects and power gesture employed to grasp larger objects. Our controllers demonstrated adaptability to the shape, size, and hardness of objects.

### 4.3 Evaluation metric

**Figure 9:**
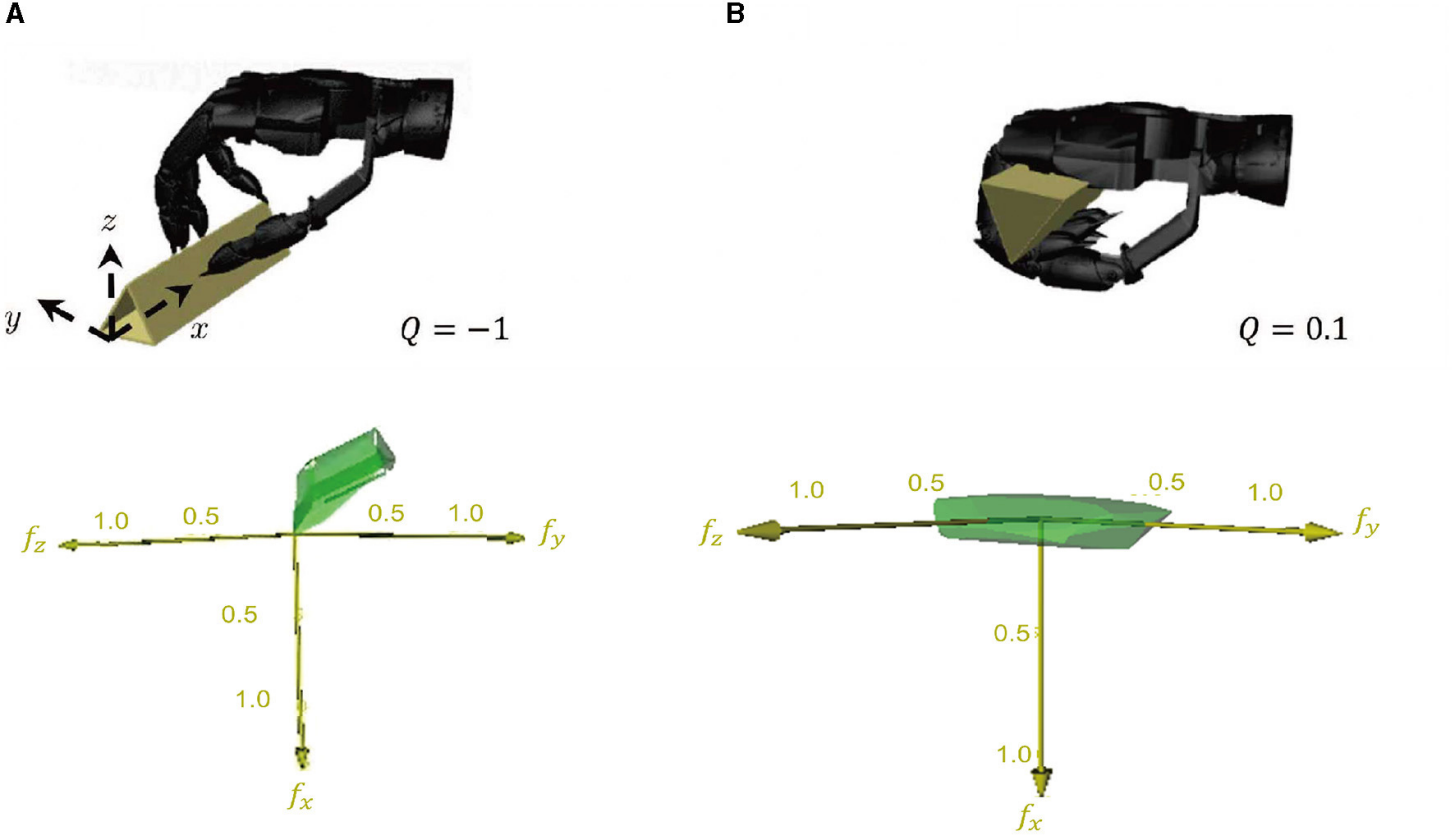
A comparison of grasping performance based on dynamics and statics. **(A)** illustrates the performance of grasp based on statics, which is not a force closure grasp. In contrast, **(B)** depicts the performance of grasp based on dynamics with a grasp quality of 0.1, which is considered a good grasp based on the metric.

To demonstrate the effectiveness of our method, a comparison is made with the quasi-statics method using the standard grasp quality metric *Q*_1_ as shown in Figure 9. A good grasp is typically defined by the force closure grasp criteria (Ferrari and Canny, 1999), which means that the applied forces and torques at the contact points can balance the external force and torque.

As an example, we considered grasping a triangular prism from a table to illustrate the advantages of our method based on dynamic processes. Using statics analysis, the contact points can only be planned on the surface of the object not in contact with the table surface. As shown in the left picture of Figure 9, force closure grasp cannot be achieved without the contact point on the contact surface between the object and the table surface. However, by considering the dynamic process of picking up a triangular prism from the table with nails, the position of the contact point can be placed on the entire surface of the object through the dynamic contact of the hand and the object, resulting in a good grasp. Our method has indeed achieved grasping a triangular prism from the table surface using the aforementioned dynamic process.

The grasp quality is significantly enhanced by the dynamic process, as indicated by the standard grasp quality metric *Q*_1_. The local grasp quality measure (LQ)


(5) $$\begin{array}{c} LQ\omega =\underset{g\in \omega A}{\max}\frac{\left\|\omega \right\|}{\left\|g\right\|} \end{array}$$

is defined as the maximum ratio between the resulting wrench *g* and applied force for a given wrench direction ω. The grasp quality measure is defined as the minimum *LQ* value over all possible wrench directions:


(6) $$\begin{array}{c} Q=\underset{\omega }{\min}LQ\omega \end{array}$$

The results of the two grasping methods are compared on the *Graspit!* simulator (Miller and Allen, 2004). The grasp using dynamic process has a grasp quality measure of 0.1, while the grasp based on the statics analysis is not a force closure grasp when the friction coefficient is less than $\frac{\sqrt{3}}{3}$. For unique objects such as triangular prisms or thin cards, the dynamic process can convert a failure of the original method into a success. For most objects, the contact point can be placed on the contact surface of the objects and the table surface via the dynamic process, resulting in an improved grasp quality.

## 5 Discussion

In this article, we propose a learning and control framework for grasping with a high DoF hand. The approach conceptualizes grasping as a detection problem, integrating deep learning with dynamic data to acquire high quality grasp. Through the incorporation of gestures, the control dimensionality is significantly reduced, reframing the challenge of high DoF hand control into the selection of a gesture and its generalized degree of freedom. The method has demonstrated generalization to objects of different shapes and successfully transferred to real robot.

Compared to methods based on static analysis, our approach provides higher grasp quality, enabling a broader range of objects, such as thin objects like cards. The results indicate that our control strategy can and achieve a success rate of over 90% for grasping objects of different sizes and shapes based on the depth image of the object, employing three hand-designed gestures.

Decoupling the selection of gestures and the choice of actions effectively addresses the challenges in controlling high DoF anthropomorphic hands. Moreover, the controller utilizes dynamic process data to explore a larger contact space on the object surface, thereby enhancing the success rate of grasping. As a general conclusion, the design of gestures and dynamic process can be considered in future research on anthropomorphic hands.

## Data availability statement

The raw data supporting the conclusions of this article will be made available by the authors, without undue reservation.

## Author contributions

SC: Conceptualization, Formal analysis, Methodology, Writing—original draft, Writing—review & editing. YJ: Conceptualization, Methodology, Writing—review & editing. HW: Funding acquisition, Writing—review & editing.
